# Viable eco-efficiency targets for waste collection communities

**DOI:** 10.1038/s41598-024-66077-y

**Published:** 2024-07-01

**Authors:** Cinzia Daraio, Simone Di Leo, Léopold Simar

**Affiliations:** 1 Department of Computer, Control and Management Engineering Antonio Ruberti (DIAG), University of Rome “La Sapienza”, Via Ariosto 25, 00185 Rome, Italy; 2 Institut de Statistique, Biostatistique et Sciences Actuarielles (ISBA), LIDAM, Université Catholique de Louvain, Voie du Roman Pays 20, B1348 Louvain-la-Neuve, Belgium

**Keywords:** Statistical methods, Data processing

## Abstract

Waste management is crucial for advancing the circular economy, and Italy has begun to address this issue by organizing municipalities into collaborative *communities of municipalities*, named ATOs. In this paper, we propose a quantitative approach based on conditional efficiency analysis to estimate *viable eco-efficiency targets* for these waste collection communities. The proposed targets are both *eco-efficient*, because they reflect optimal resource allocation within the eco-efficiency framework, and *viable*, because they consider the unique specificities of each waste community. The methodology determines a pathway or direction for municipalities to reach the eco-efficiency frontier based on specific external factors, ensuring that each municipality is benchmarked against others with similar contexts within the same community. Our analysis focuses on 89 Italian municipalities within the *ATO “Città metropolitana di Roma Capitale”* in 2021, revealing that *size* and *economic development* significantly contributed to *viable eco-efficiency* within the community during this period. The proposed approach is general and flexible and can be applied to other municipalities in Italy or across Europe. It can also be extended to meso (regional) or macro (country) levels of analysis.

## Introduction

In an era characterized by unsustainable consumption patterns, the traditional linear economy based on the “take, produce, dispose” paradigm is becoming increasingly unsustainable, due to the overexploitation of resources and the production of large amounts of waste. To address these issues, many countries are shifting towards a circular economy (CE) model. Although there is no universally accepted definition of the CE, one of the most precise definitions was proposed by^1^, describing it as “an economic system that replaces the ‘end-of-life’ concept with reducing, alternatively reusing, recycling, and recovering materials in production/distribution and consumption processes.”

The CE transition requires attention to waste and its management. This has been recognized by the United Nations Development Program, which, in 2015, included waste management in its Sustainable Development Goals (SDGs, https://sdgs.un.org/). The United Nations’ focus on this topic has catalyzed further attention from both developed countries (e.g. Canada^2^, Mexico^3^, Taiwan^4^) and developing countries^5–7^, highlighting the complexities involved in adopting CE principles^8^. The European Union (EU) has also taken action, introducing rules as early as 2008 to accelerate the transition^9^. The EU’s focus on the CE is linked to two goals, as “the transition to a circular economy is essential to achieving our climate and environmental goals and creating a more competitive and sustainable economy.”^10^.

The advent of rules geared towards a CE transition has resulted in numerous studies on the efficiency of municipal waste (MW) management in various European countries, including Belgium^11^, Poland^12^ and Germany in the north^13^, and Spain^14^, Italy (see^15^ and “State of the art”) and Portugal in the south^16^. Individual country studies at the European level are necessary, due to the complexity of MW management, which is organized differently across countries (for further detail, see^17^). Research into the efficiency of MW collection is essential for advancing the CE transition. This is because waste collection is an important and expensive public service worldwide, generally accounting for 75–80% of MW management budgets^18^. Improved efficiency in this sector could free resources for waste disposal or other municipal expenditures. Moreover, as noted by^19^, effective waste management increases financial performance by lowering costs while providing environmental benefits. In this vein, efficient MW collection is a central goal of many countries.

Efficiency, in general, is assessed by comparing the output/input ratio of a given unit against that of a benchmark^20^. The more specific concept of “eco-efficiency”^21,22^ involves the production of more goods and services with fewer resources and minimal environmental impact. The focus on eco-efficiency in MW collection derives principally from the need to minimize waste collection costs and reduce social and environmental impacts, promoting greater system efficiency and fairness^23^. Additionally, effective separate waste collection and recycling can generate significant benefits, such as resource savings, reduced emissions and job creation^24^. The *eco-efficiency* concept relates mainly to two pillars of sustainability—economics and *environmental protection*—while often excluding social factors (as these are not under a municipality’s direct control). However, social factors can nonetheless significantly influence efficiency, and they are therefore relevant “external factors.”

There are two main methods for estimating efficiency. The parametric approach assumes a specific functional form linking inputs to outputs and a defined functional form for the efficient or best practice frontier. It also includes a measure of inefficiency (i.e., the distance between an observation and the efficient frontier). In contrast, the non-parametric approach does not assume a particular functional form for the efficient frontier or for inefficiency. Data envelopment analysis (DEA), a widely used non-parametric method, employs linear programming to construct the efficient frontier by enveloping the observed data^25^ (see “Materials and methods” for further detail).

The main objective of this paper is to propose a quantitative approach based on conditional efficiency analysis. This method incorporates “external factors” into the assessment of efficiency, providing a more contextualized evaluation (see^20^). Our approach aims at estimating *viable eco-efficiency targets* for waste collection communities. These targets are *eco-efficient* because they are derived from an optimal allocation of resources within an eco-efficiency framework, and they are *viable* because they consider the specific characteristics of each waste community. We present new empirical evidence on eco-efficiency in Italian waste management by applying conditional data envelopment analysis^26^ and its recent extension, which uses a flexible path (i.e., so-called directional distance) to reach the efficient frontier^27^. The “*viable eco-*efficiency” targets we propose are defined as combinations of inputs and outputs that make a municipality efficient, considering its specific external/contextual factors. This directional approach to reaching the efficient frontier makes our targets more realistic and “feasible” than conventional targets.

The primary contribution of the paper is the introduction of a quantitative approach that incorporates external factors into the “eco-efficiency” estimation, providing policymakers with operational targets that consider both output/inputs metrics and contextual factors. To demonstrate the effectiveness of this approach, we analyze 89 Italian municipalities within the *Area Territoriale Ottimale* (ATO) ‘Città Metropolitana di Roma Capitale’ (ATO-RC) for the year 2021. This context is particularly noteworthy due to the significant heterogeneity of the municipal communities involved, in terms of the external factors that influence their waste collection processes, raising questions about the equity and sustainability of performance comparisons.

The article is structured as follows: “State of the art” introduces the state of the art on efficiency in the MW sector; “Materials and methods” introduces the materials and methods used; “Results and discussion” presents the main results; and “Conclusions” discusses the findings and the main limitations of the study, while providing concluding remarks.

## State of the art

Waste collection represents a significant expense for municipal budgets^18^. Research on the efficiency of MW has explored this topic from multiple perspectives, with a range of objectives. For example, studies have investigated the impact of privatization policies on waste management^28^, the effects on collection processes^3^ and the regulatory impact on efficiency^29^, using different methods and levels of analysis. Four levels of analysis have been recognized in the MW domain: the macro (i.e., country) level, the meso (i.e., regional) level, the micro (i.e., municipal or local utility-based) level and individual case studies (of, e.g., cities). Macro-level analyses have demonstrated that effective waste management can facilitate the CE transition^30–32^ and identified strategies for countries to improve their situations^33^. At the meso level, research has typically focused on waste disposal^34^ and waste management^35,36^. Additionally, interesting case studies have provided evidence to improve situations, particularly for cities characterized by complex geographical or social structures^37–39^.

While meso- and macro-level studies provide broad insights, they often lack sufficient specificity to set goals and targets for individual municipalities. On the other hand, while case studies can be helpful, they may not be easily applicable to other contexts. For this reason, micro-level analyses have attracted considerable interest. Adding further support for this approach, in many countries, the responsibility for MW collection falls to municipalities.

At a micro level, various methods have been proposed to assess waste management efficiency. According to a review by^40^, these methods can be classified into three main groups: data generation methods (e.g., surveys); simple evaluation methods that compare performance against benchmarks (offering an initial overview but lacking depth); and complex evaluation methods such as life cycle assessment (LCA), multi-criteria decision-making (MCDM) and data envelopment analysis (DEA). LCA^41^ evaluates the environmental impact of a product or service throughout its life cycle. MCDM methods (e.g., ELECTRE^42^ or the analytic hierarchy process^43^) enable the comparison of different alternatives and the selection of the best option based on specified objectives. However, it is important to note that the choice of criteria and their assigned weights can be subjective, potentially affecting the results. In contrast, DEA is a non-parametric method that assesses the efficiency of a decision-making unit (DMU) against a set of similar DMUs. DEA is the most widely used method for analyzing efficiency in the literature^44^. It has the advantage of being adaptable to various contexts, as it does not require any a priori assumptions about the shape of the production function or the distribution of inefficiency. However, DEA can be sensitive to the choice of inputs and outputs, as well as to outliers. Furthermore, unlike MCDM, DEA is based on empirical data and does not rely on subjective evaluations of criteria or weights.

A critical aspect of efficiency assessment in waste management concerns the evaluation of external factors. Commonly, researchers have used DEA to incorporate relevant external factors^45,46^. In this paper, we apply directional DEA (D-DEA), representing a variant of DEA that offers greater flexibility in measuring efficiency. Standard DEA is radial, implying an equiproportional reduction of inputs with outputs maintained as constant (i.e., input-orientation)^44^ or an equiproportional expansion of outputs given fixed inputs (i.e., output-orientation)^36^. In contrast, D-DEA measures efficiency according to a specific path towards the efficient frontier, defined by a direction vector (see “Methods”) D-DEA is particularly flexible, allowing for simultaneous increases in some outputs (e.g., recycling) and reductions in others (e.g., untreated waste), making it well-suited for the *eco-efficiency* framework considered here (see, e.g.,^45,47,48^).

This paper focuses on the context of Italy—a country with long-standing challenges in MW collection and unique regulations that have created *communities of municipalities* (ATOs) to make collective decisions on MW management^49^. There is a rich literature on MW management efficiency in Italy that proposes several relevant external factors. Table 1 summarizes the main external factors identified in the literature for the Italian context, grouped into three categories: morpho-demographic factors, socio-economic factors and technical factors. Our analysis focuses on the *community of municipalities* within the *ATO “Città metropolitana di Roma Capitale*” (ATO-RC), located in the Latium region of central Italy, near Rome. Previous studies have often focused on specific regional contexts, such as Apulia^50–52^, Tuscany^53,54^, Campania^55^, Abruzzo^25^ and Verona^56^. To the best of our knowledge, ours is the first study to examine MW management efficiency in the Latium region.

**Table 1:** Main external factors identified in the literature for the Italian case. Factors are grouped into three categories, according to the nature of the factors considered.

Morpho-demographic factors	Socio-economic factors	Technical factors
Altimetry^25,35,60,70^	Per capita income and total income^25,71,72^	Road length and city size^25,50^
Population served and population density^15,50,53,56,60,73–75^	Population seniority^53^	Collection method^56^
Geographical location^71,75,76^	Corruption institution quality and criminal activities^35,72,76–80^	Load capacity and vehicle usage^56,81^
Household characteristics^25,56^	Population education^73^	
	Tourist and non-residence presence^25,56,81^	

The uniqueness of ATO-RC lies in its position in central Italy, which bridges the north–south divide in terms of economic and social indicators. Central Italy shares more similarities with northern Italy than southern Italy, particularly with respect to the SDGs^57,58^. However, many studies on Italian waste management have failed to adequately consider how external factors might influence not only the efficiency frontier, but also municipal efficiency targets. Thus, there is a gap in the literature related to the use of D-DEA to estimate eco-efficiency, considering external factors. Additionally, ATO-RC has not yet been studied in detail.

## Materials and methods

### Data

Our study focused on the MW collection of 89 Italian municipalities within ATO-RC, in 2021. The year 2021 was chosen for the analysis because it related to the most recent set of consolidated data available.

The context of ATO-RC, which encompasses 89 municipalities with a total population of 1,277,177 inhabitants, provided an excellent basis for calculating *viable eco-*efficiency targets within a community of municipalities. Furthermore, the analysis of municipalities within a single ATO, rather than across different ATOs, enabled more meaningful local insights to be gained and prevented misleading aggregated analyses that might not fail to meet policymaker needs. Of note, the municipality of Rome (part of another ATO for waste collection) and municipalities within the *Aniene Valley consortia* were excluded from the analysis.

To estimate *eco-efficiency*, we included the total cost of waste collection per capita as the input, the tons of unsorted waste as the bad output and the tons of sorted waste as the good output. Additionally, we considered two external factors that might influence eco-efficiency: municipality *SIZE* and *RICHNESS*.

Data were obtained from the *Istituto Superiore per la Protezione e la Ricerca Ambientale* (ISPRA)^59^ report on MW. The dataset included the total cost of MW collection per capita, the tons of sorted MW and the tons of unsorted waste in each municipality. To assess the efficiency of municipalities in balancing collection costs and waste collection, we used a model that considered the total cost of waste collection per capita as an input (measured in euros per capita, hereafter referred to as “X”), the tons of unsorted waste as a *bad* output (hereafter referred to as “BY”) and the tons of sorted waste as an output (hereafter referred to as “Y”). The bad output (BY) was treated as an input in the analysis because municipalities aim at reducing it, rather than increasing it (in contrast to normal outputs). This model, which aligns with the *eco-efficiency* framework, has already been proposed in previous studies^25^, sometimes with slight variation^60^.

**Figure 1:**
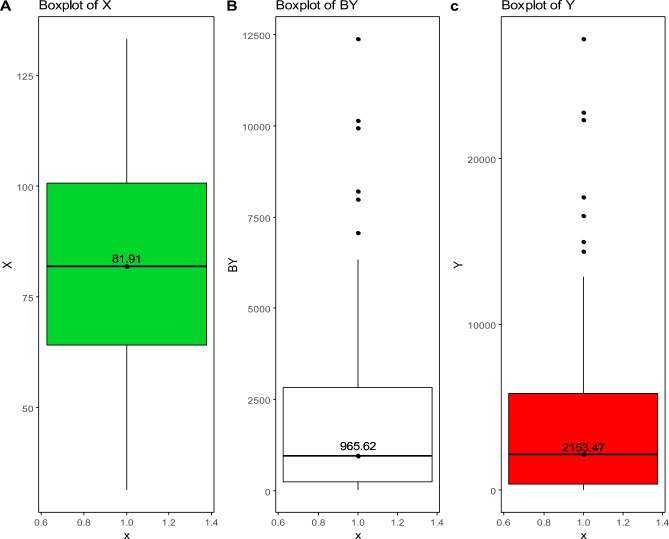
Boxplots of the input (total cost of waste collection per capita, X, in green, Panel (**A**)), bad output (tons of unsorted waste, BY, in white, Panel (**B**)) and output (tons of sorted waste, Y, in red, Panel (**C**)) considered in the analysis. Numbers reported in the boxplots represent the medians.

**Figure 2:**
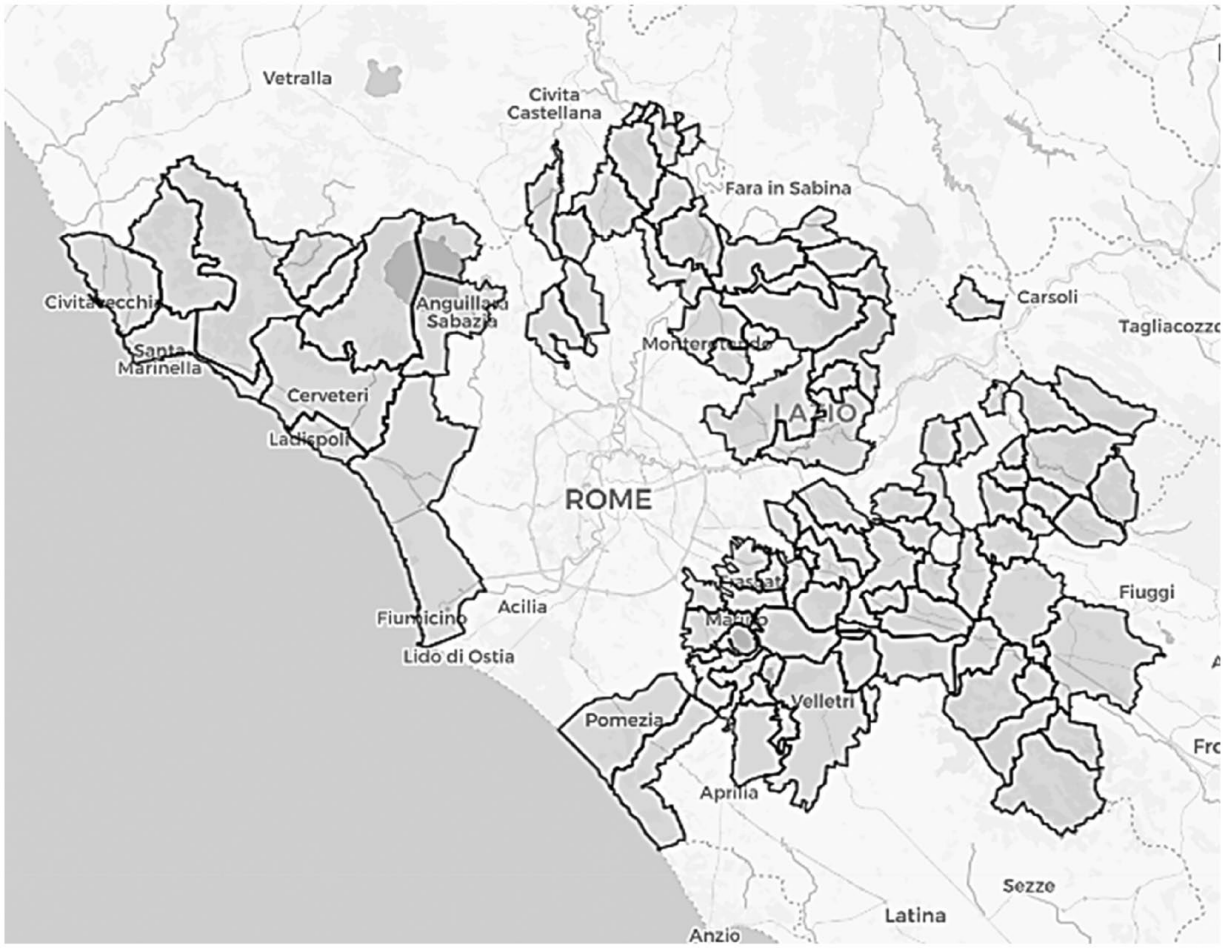
Map of the 89 municipalities considered in the analysis. The municipalities considered are colored in grey and delimited by a black line. Source: Own elaboration, generated using R (version 4.3.2) and the Leaflet R package (version 2.2.1) on OpenStreetMap map (https://openstreetmap.org) and CartoDB tiles (https://cartodb.com/attributions).

In the analysis, all variables were scaled by their empirical standard deviation to improve the calculations. Figure 1 presents the boxplots for the input (total cost of waste collection per capita, X, in green), the *bad* output (tons of unsorted waste, BY, in white) and the output (tons of sorted waste, Y, in red). Figure 2 shows a geographical map of the municipalities considered, highlighted in grey.

**Figure 3:**
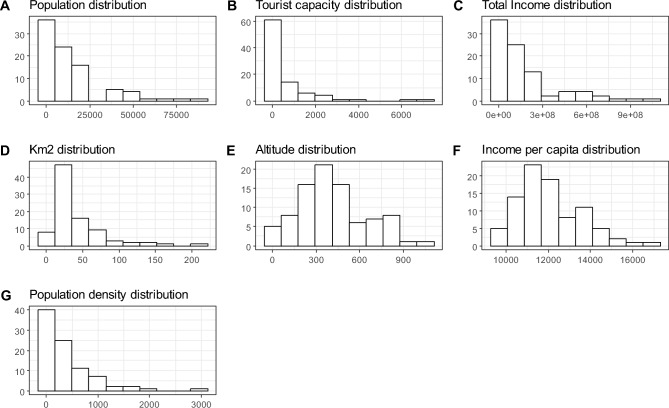
Histograms of the external factors considered.

Key characteristics and external factors for the 89 ATO-RC municipalities were gathered from the ISTAT database^61^. The data included information on the population served, land area (in km^2^), population density, taxable income (considering the financial year preceding the year of analysis), taxable income per capita, altitude and tourist capacity. Figure 3 shows the histograms of these external factors for all 89 municipalities. Due to the relatively small sample size and the non-parametric nature of our method, including all of the collected factors in the analysis could have led to the so-called “curse of dimensionality.” To address this issue, we used principal component analysis (PCA, see “Methods” for further detail) to reduce the number of external variables, prior to conducting the DEA analysis. Specifically, we aggregated the original variables into two principle components. The first component, which we called SIZE, was highly correlated with population, touristic capacity and territorial dimension, and we included it as our first external variable (Z1). The second component, which we called RICHNESS, was strongly correlated with taxable income per capita, and we included it in our analysis as our second external variable (Z2). Table A1 provides further detail on these correlations and their components.

### Methods

D-DEA is a method used in efficiency analysis to evaluate the performance of a unit along a specific path towards the efficient frontier. This is done by specifying a directional distance (see, e.g.,^62^). Directional distance-based measures rely on directional distance functions (DDFs), which offer a flexible approach to measuring unit inefficiency. In mathematical terms, DDFs work as follows. Given a production set defined as:

$$\Psi = \{ \left( {{\text{x}},{\text{y}}} \right) \in {\text{R}}_{ + }^{{{\text{p}} + {\text{q}}}} |{\text{x}} \,can \,produce \,{\text{y}}\}$$

where $x\in {R}_{+}^{p}$ is the vector of inputs, $y\in {R}_{+}^{q}$ is the vector of outputs,* (x,y)* represents a combination of a unit’s (in our case a municipality) inputs and outputs, and $\Psi$ is the true but unknown production set. The directional distance measure is defined as

$$\delta \left( {x, y} \right) = \sup \left\{ {\delta {|}\left( {x{-}\delta d_{x} ,y + \delta d_{y} } \right) \in \Psi } \right\}$$

where $\delta$ is the efficiency score, and d_x_ ∈ ${R}_{+}^{p}$ and d_y_ = ${R}_{+}^{q}$ are the direction vectors that define the path to the efficient frontier. Therefore, the distance is measured along a path determined by the direction vector $d^{{^{\prime } }} = \left( { - d^{{^{\prime } }} x, d^{{^{\prime } }} y} \right)$ in an additive way, whereby if *(x, y)* lies on the efficient frontier, then $\delta (x, y)=0$. In practice, directional efficiency measures quantify the extent to which a unit can reduce inputs or increase outputs while maintaining the same proportion among them, in a specific direction. These measures are particularly useful when the units under analysis operate in diverse contexts that may affect their combinations of inputs and outputs. A key challenge of DDF is the determination of the appropriate direction for analysis. Daraio and Simar (2016)^27^ proposed a data-driven approach to address this challenge, based on the external factors that characterize the unit. To estimate the conditional direction (i.e., the direction towards the efficient frontier based on external environmental variables),^27^ outlined three main steps:

1. Convert each observation into a p + q-dimensional input/output matrix ${X}_{i}{Y}_{i}$ in polar coordinates $({r}_{i}, {\theta }_{i}), for i=1, \dots , n$, where r_i_ > 0 and ${\theta }_{i}= ({\theta }_{i}1 , \dots , {\theta }_{i}^{p+q-1})$ (in our case, p + q = 3).
2. Perform a polar non-parametric regression for each component θ^j^ on W (i.e., the matrix of external factor $W\in {R}^{d}$) to estimate $E\left({\theta }^{j} |W\right)$, considering ${\theta }^{j} , j=1, \dots , p +q - 1$. For this, use for each regression j, j = 1, …, p + q − 1 the set of data $\left({\theta }_{i}^{j} |{W}_{i}\right)$, i = 1, …, n (using as bandwidth selection for each regression the cross-validation method).
3. Convert to Cartesian coordinates the directional vector *d f*rom the polar coordinates (${r}_{i}; \widehat{{\theta }_{i}})$, to obtain $\widehat{d}=\left({r}_{i}; \widehat{{\theta }_{i}}\right).$

In the present study, these steps were used to generate a derived directional vector $\widehat{d}$ to estimate efficiency scores, using DEA variable returns to scale. Subsequently, efficiency targets were calculated on the basis of the efficiency scores $\widehat{\delta }$ for each municipality, using the following equation:

$$Targets: \left( {x{-}\hat{\delta }\hat{d}_{x} ,y + \hat{\delta }\hat{d}_{y} } \right)$$

with $x{-}\hat{\delta }\hat{d}_{x}$ representing the input targets and $y + \hat{\delta }\hat{d}_{y}$ representing the output targets.

To determine the optimal direction, we considered external factors that might influence waste collection, such as population served, land area (in km^2^), population density, taxable income, taxable income per capita, altitude and tourist capacity. To address the “curse of dimensionality” (common in non-parametric methods applied to small sample sizes), we used PCA (for an introduction, see^63^) to reduce the dimensionality of the external factors.

PCA is a statistical technique that transforms a set of correlated variables into a smaller set of uncorrelated variables (called “principal components”), effectively reducing dimensionality. It works by calculating the variance-covariance matrix, which contains the variances and covariances for each pair of variables, and then diagonalizing this matrix using an orthogonal transformation. This process identifies the principal components and orders them by their explained variance, with the first component capturing the most significant variance in the data. PCA allows for dimension reduction by selecting only the *k* most informative principal components, where *k* represents the desired number of dimensions. In the present study, we applied PCA to five variables: population, tourist capacity, income per capita, land area (km^2^ in log scale) and altitude. We did not incorporate total income and population density, due to their high correlation with population (correlation coefficients of 0.99 for total income and 0.85 for population density). The variables were log-transformed due to their distribution (see Fig. 3). Appendix A reports details of the PCA results, including the relationship between the calculated components and the original variables.

## Results and discussion

**Figure 4:**
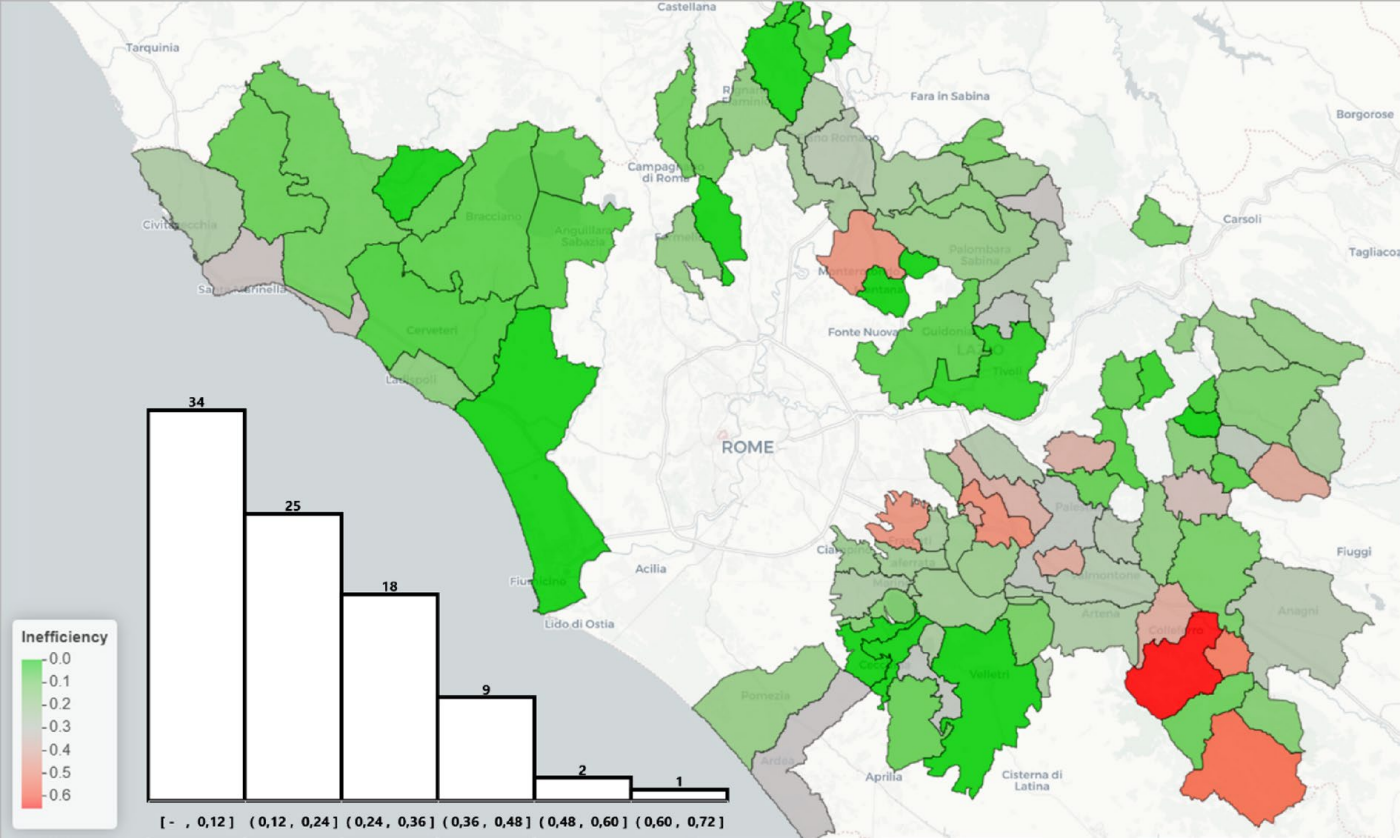
Map and distribution of the infficiency scores of the municipalities analyzed. Colors indicate the inefficiency value, ranging from 0 (efficiency, in green) to 0.65 (inefficiency, in red). In the histogram of the inefficiency scores, the number of observations included in each histogram bar is reported above. Source: Own elaboration, generated using R (version 4.3.2) and the leaflet R package (version 2.2.1) on OpenStreetMap map (https://openstreetmap.org) and CartoDB tiles (https://cartodb.com/attributions).

We estimated the efficiency scores for all ATO-RC municipalities using the methods described in the previous section. Efficiency scores can range from zero (indicating total efficiency) to infinity (indicating total inefficiency). In the present analysis, the average inefficiency score (greater than zero) was 0.19. Notably, only 10 municipalities demonstrated full efficiency, indicating room for improvement across the community. Figure 4 presents a histogram of the inefficiency scores and a map showing their geographical distribution. In the map, green represents efficient municipalities, while red delineates inefficient municipalities. The geographical pattern suggests that coastal municipalities tended to be more efficient, on average, compared to those located inland.

Efficiency gaps were estimated using the conditional efficiency methodology described above, in which the direction for reaching the efficient frontier was defined by the two external factors SIZE (Z1) and RICHNESS (Z2) (efficient municipalities had gaps equal to 0). These gaps represented the *“viable eco-efficiency”* targets that inefficient municipalities should aim for to achieve efficiency, based on the performance of *“peer”* municipalities with similar contexts.

**Figure 5:**
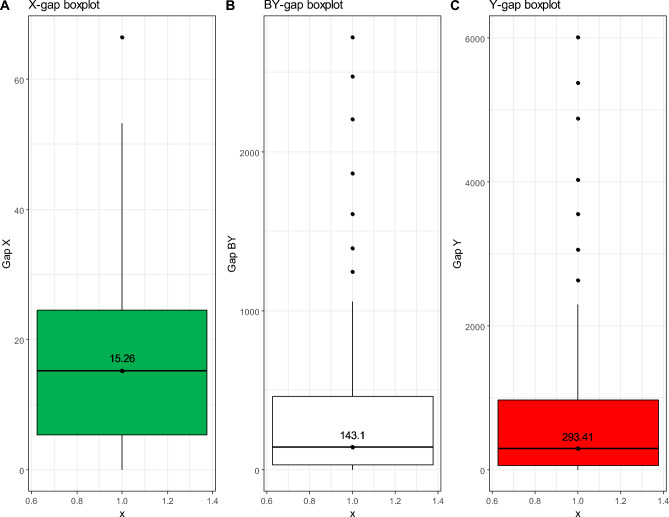
Boxplots of the input and output gaps of the inefficient units. The values reported are in the original scale (X in euros per capita, BY and Y in tons). The numbers in the boxplots are the medians.

Figure 5 illustrates the boxplots of the efficiency gaps for the ATO-RC municipalities. The median values suggest that, for a typical municipality to become efficient, it should: reduce waste collection costs by 15.26 euro per capita (X; 19% reduction), decrease unsorted waste by 143.1 tons (BY; 15% reduction) and increase sorted waste by 293.41 tons (Y; 14% increase).

**Figure 6:**
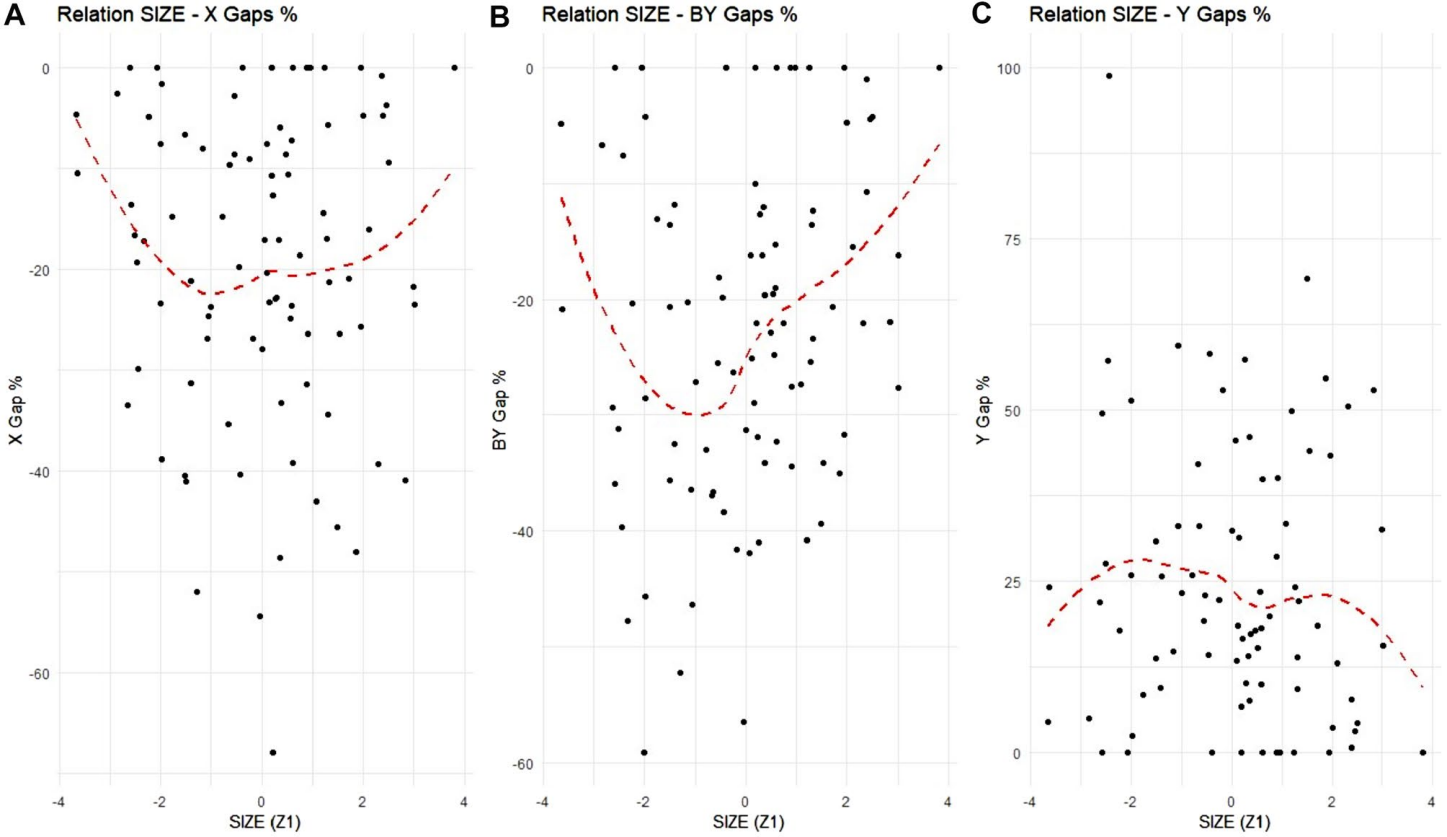
Gaps of X (Panel **A**), BY (Panel **B**) and Y (Panel **C**) of each municipality about SIZE (Z_1_). Each point represents a gap in percentage value of a municipality in ATO-RC. The red dotted curve represents a LOESS (locally estimated scatterplot smoothing) with smoothing at 0.75 and polynomial degree 2. Note that, in Panel C, there are several outlier municipalities (some not reported).

Figure 6 illustrates the relationship between the efficiency gaps for the input (X), bad output (BY) and output (Y) in percentage terms, relative to municipality SIZE. Each point represents a municipality, showing considerable variability in the data. Despite this, some trends can be observed. In Panel C, there appears to be an *inverted U-shaped relationship* between the inefficiency in output production (measured by the Y gaps in percentage) and municipality SIZE. Initially, as SIZE increases, there is an *increasing* trend in the Y gaps, indicating growing inefficiency in output production. However, beyond a certain point, the Y gaps in percentage begin to *decrease* for municipalities of greater SIZE, suggesting lower inefficiency for larger municipalities.

Panel A depicts the relationship between the inefficient usage of the input X (total cost of waste collection per capita, in percentage) and municipality SIZE, following a *U-shaped pattern*. Initially, as SIZE increases, the X gaps in percentage are negative, indicating a reduction in inefficient usage of the input. However, as SIZE continues to increase, the X gaps in percentage begin to rise, suggesting more inefficient usage of inputs, before eventually *decreasing* for larger municipalities. A similar U-shaped trend can be observed in Panel B, which shows the inefficient production of the bad output (BY, tons of unsorted waste, in percentage). As in Panel A, this graph initially shows a negative trend as SIZE increases, followed by a rise in inefficiency and then a decline for larger municipalities.

**Figure 7:**
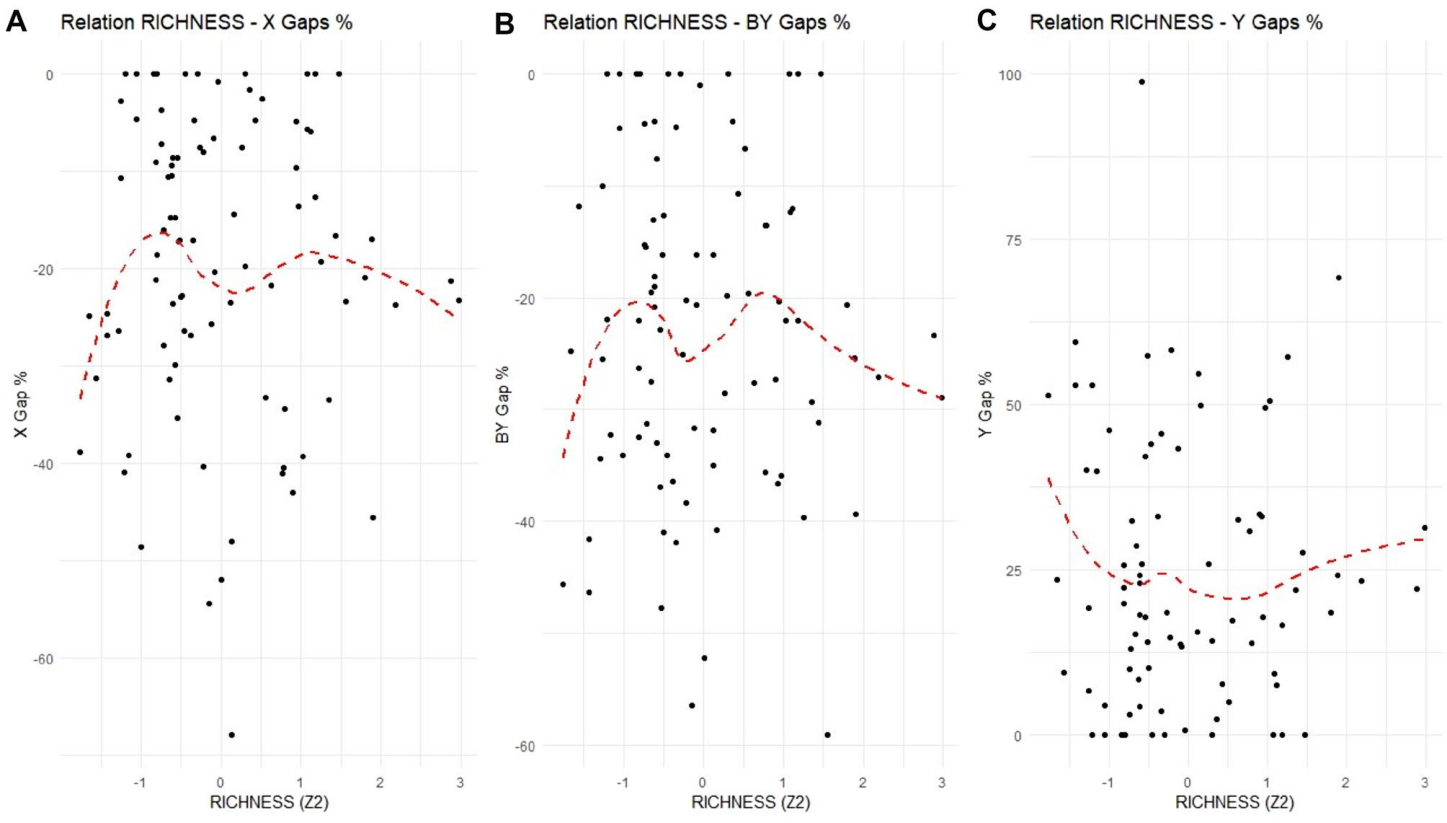
Gaps in percentage of X (Panel **A**), BY (Panel **B**) and Y (Panel **C**) of each municipality to RICHNESS (Z_2_). Each point represents a gap in percentage value of a municipality in ATO-RC. The red dotted curve represents a LOESS (locally estimated scatterplot smoothing) with smoothing at 0.75 and polynomial degree 2.

Figure 7 depicts the relationship between the efficiency gaps for the input (X), bad output (BY) and output (Y) in percentage terms, relative to municipality RICHNESS. As in the previous figure, the points (representing municipalities) are widely spread, indicating high variability in the data. However, there is a noticeable pattern, where RICHNESS seems to exhibit the opposite behavior compared to SIZE. In Panel C, there is a *U-shaped relationship* between RICHNESS and inefficiency in output production, measured by the Y gaps in percentage. Initially, as RICHNESS increases, there is a *decreasing* trend in the Y gaps, indicating lower inefficiency in output production. However, beyond a certain point, the trend reverses, with the Y gaps increasing in line with RICHNESS. A similar U-shaped pattern may be observed in Panels A and B, which illustrate the relationship between RICHNESS and the inefficiency in input use (X gaps, in percentage) and bad output production (BY gaps, in percentage).

These results suggest that *scale* and *economic development* play critical roles within this waste collection community, and that municipalities could improve their efficiency by achieving an optimal size to benefit from economies of scale. This relationship between scale and economic development in waste collection has been analyzed in previous studies^53,56^.

To illustrate the relevance and potential of *viable eco-efficiency* targets in the fair benchmarking of municipalities, considering their specific contexts, we will present two case studies of the municipalities of Ciampino and Guidonia Montecelio. Ciampino, a municipality of almost 39,000 residents, is home to the low-cost Rome-Ciampino *"G. B. Pastine"* airport. This airport is one of the busiest in Italy, registering more than 2,300,000 passengers in 2021^64^). According to our analysis, Ciampino has an efficiency score of 0.21.

Using the approach proposed by^27^ (see the "Methods" section for more detail), we identified the efficiency target that Ciampino must achieve to reach optimal performance. This approach may help policymakers recognize Ciampino’s best-performing *peer*, or “benchmark,” represented by an efficient municipality with a similar input–output mix and comparable external factors. While multiple benchmarks may exist, the method proposed by^27^ identified Albano Laziale as the most suitable benchmark for Ciampino. Albano Laziale is a city with 39,466 residents, and one of the most important municipalities in the *Castelli Romani* zone.

The second case study focuses on Guidonia Montecelio, representing the largest municipality in our dataset by population (88,237 residents), with an efficiency score of 0.06 (6% inefficient). Also in this case, we applied the same benchmark identification process, which pointed to Velletri as the most suitable benchmark. Velletri, located north of Rome, has 52,151 residents, making it the most populous municipality of the *Castelli Romani* zone.

**Figure 8:**
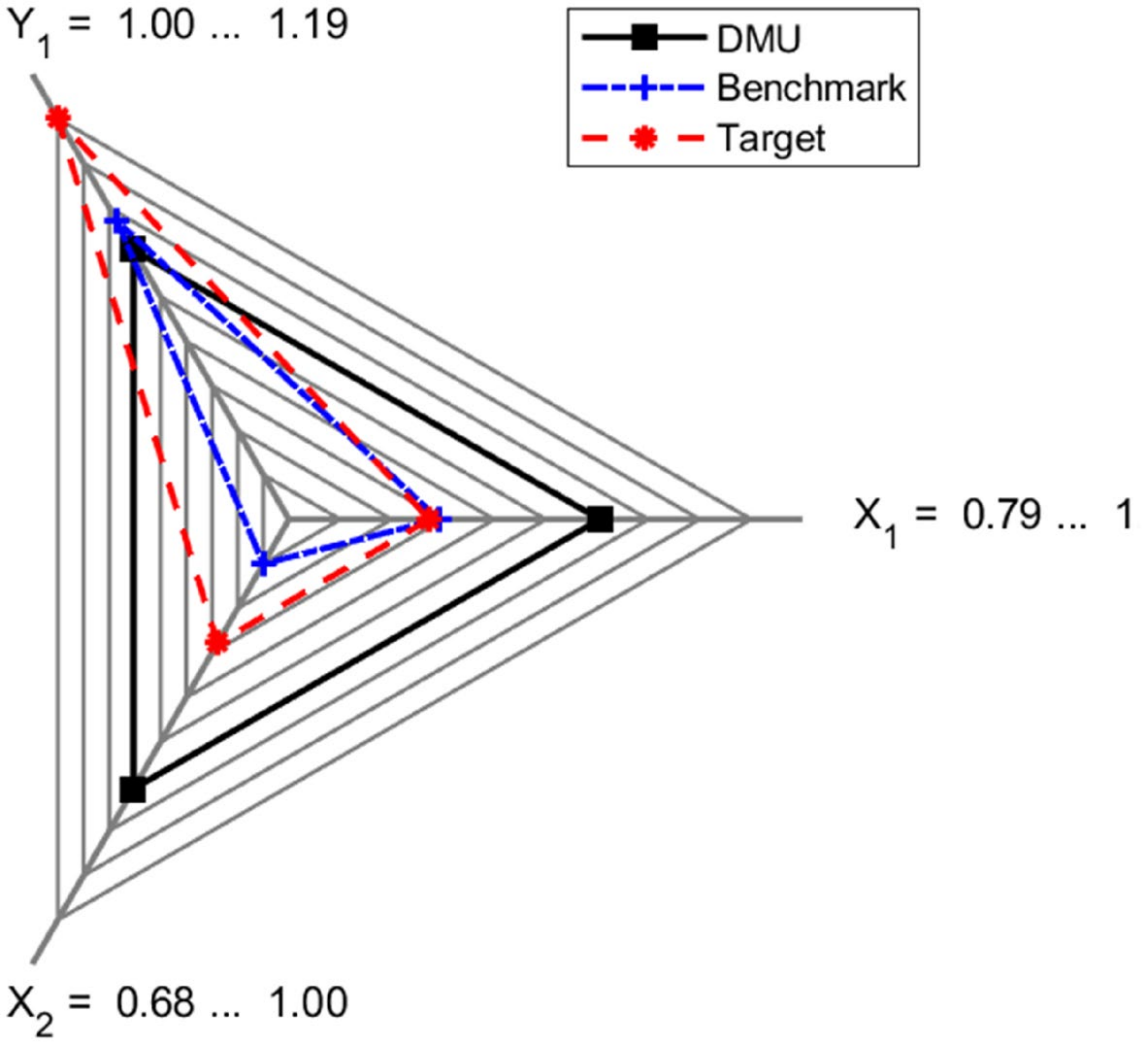
Input(X_1_), bad output (X_2_) and output target (Y_1_) for Ciampino to reach efficiency. In red, target values are presented. In blue, the benchmark (Albano Laziale) values are presented. In black, the original Ciampino data are presented. The data are scaled based on the maximum and minimum DMU, benchmark and target values. The X_1_ axis represents the X values, the X_2_ axis represents the BY values and the Y_1_ axis represents the Y values.

**Figure 9:**
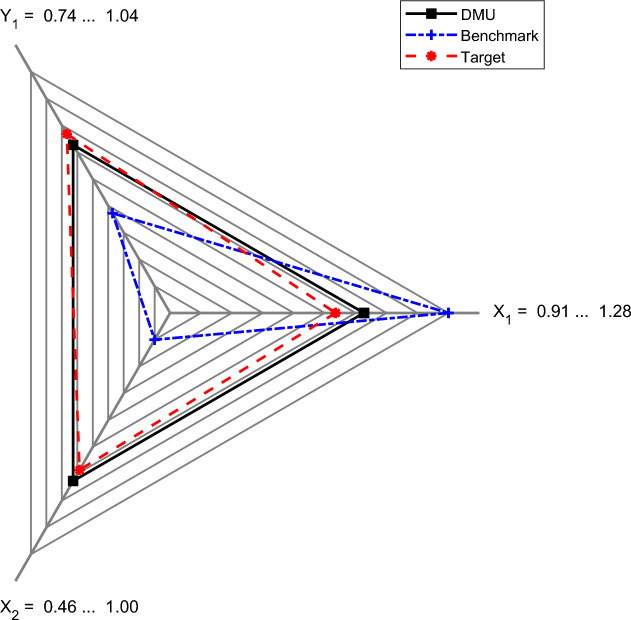
Input (X_1_), bad output (X_2_), and output (Y_1_) target for guidonia montecelio to reach efficiency. In red, target values are presented. In blue, the benchmark (Velletri) values are presented. In black, the original guidonia montecelio data are presented. The data are scaled based on the maximum and minimum DMU, benchmark and target values. The X_1_ axis represents the X values, the X_2_ axis represents the BY values and the Y1 axis represents the Y values.

Figures 8, 9 present radar plots illustrating the percentile contraction of inputs (with the X_1_ axis representing collection costs per capita and the X_2_ axis representing unsorted waste) and the expansion of output (represented by the Y_1_ axis for sorted waste) required for Ciampino (Fig. 8) and Guidonia Montecelio (Fig. 9) to become efficient.

In these figures, the original values for the analyzed municipalities (Ciampino in Fig. 8 and Guidonia Montecelio in Fig. 9) are labeled as “DMU” (i.e., decision making unit) and shown in black. The benchmark values (Albano Laziale in Fig. 8 and Velletri in Fig. 9) are displayed in blue, while the efficiency targets (i.e., the desired values for each municipality to achieve efficiency) are indicated in red. These figures may help to focus the efforts of managers and policymakers to improve municipal performance. Table 2 provides the numerical values for both cases. For Ciampino, the results (in Fig. 8 and the first half of Table 2) indicate that policymakers should concentrate on a 21% reduction in both the total cost of waste collection per capita (X) and the tons of unsorted waste (BY), while aiming for a 16% increase in sorted waste (Y). This is because the benchmark, Albano Laziale, has lower collection costs (93.87 euros per capita vs. 117.06 euros per capita in Ciampino) and a higher rate of waste separation (79%, compared to Ciampino’s 71%). To become efficient, Ciampino should therefore aim at reducing collection costs to 92.54 euros per capita and increasing waste separation to achieve a rate of 78%.

**Table 2:** Values of inputs, output and Zs for the two case studies. The table is divided into two parts. The first part concerns case study 1 on Ciampino, Albano and the viable targets to be reached for Ciampino. The second part concerns case study 2, on Guidonia Montecelio, Velletri and the viable targets to be reached for Guidonia Montecelio. X is in euros per capita, and BY and Y are in tons.

Municipality	X	BY	Y	Population	Income per capita
Case study 1: Ciampino					
Albano laziale (benchmark)	93.87	3486.34	12860.55	38589	14577
Ciampino	117.06	5117.24	12354.17	39466	13513
Viable target (for ciampino)	92.55	4061.22	14645.84	–	–
Case study 2: Guidonia montecelio					
Guidonia montecelio	64.16	10129.95	22361.13	88237	11904
Velletri (benchmark)	82.10	4669.40	16534.52	52151	11055
Viable target (for guidonia montecelio)	58.15	9702.47	23301.43	–	–

With regard to Guidonia Montecelio (see Fig. 9 and the second half of Table 2), this municipality outperforms its benchmark in terms of collection costs, spending 64.16 euros per capita compared to 82.10 euros per capita for Velletri. However, Guidonia Montecelio falls short in waste sorting, with only 69% sorted waste collection, compared to 78% for its benchmark. Given its efficiency score of 0.06, Guidonia Montecelio requires only small adjustments to achieve efficiency: reducing its costs by 7%, to achieve a value of 58.15 euros per capita; decreasing the amount of unsorted waste by 4%, to achieve a value of 9,702.47 tons; and increasing sorted waste collection by 4%, to achieve a value of 23,301.43 tons. These actions would lead to a 71% waste separation rate for Guidonia Montecelio.

While these targets are helpful for determining the necessary actions for achieving efficiency, the question of how Guidonia Montecelio and Ciampino might achieve them, remains. Despite the common use of “door-to-door” waste collection methods across these municipalities and their benchmarks, the noted differences in inefficiency may be influenced by other factors, such as the choice of waste collection operator. In this sense, it is interesting to note that both benchmarks (Albano Laziale and Velletri) share the same waste operator (Volscambiente, https://www.volscambiente.it/raccolta-differenziata/), suggesting that operational practices could play a role in achieving efficiency. Beyond this, Guidonia and Ciampino might also explore the implementation of needs-based collection based on intelligent sensors that monitor waste can fill levels and schedule collection accordingly, or waste tax discounts to residents who reduce waste or recycle properly. However, in our opinion, the key actions are collaboration and the sharing of best practices, not only among state entities but also among waste collector operators. Crucially, the achievement of efficiency targets does not require immediate and drastic change. Rather, knowledge of a municipality’s position and inefficiency, along with the identification of a comparable benchmark and target values, lays the foundation for learning and improvement.

## Conclusions

The transition to the CE is increasingly gaining recognition as essential for ensuring long-term sustainable progress. Waste management and collection play pivotal roles in this transition, and Italy has taken strides to address waste management challenges by establishing collaborative *communities of municipalities* (ATOs).

In the present study, we proposed a quantitative approach grounded in conditional efficiency analysis to estimate *viable eco-efficiency targets* for waste collection communities within an eco-efficiency framework. The identified targets are both *eco-efficient*, because they optimize resource allocation, and *viable*, because they consider the external factors that influence waste collection efficiency. Our estimation of these targets incorporated directionality conditioned by external factors.

Our study focused on the *ATO “Città metropolitana di Roma Capitale*,*”* comprised of 89 municipalities. This ATO has never been explored in previous research. Building on the literature, our model for estimating the eco-efficiency of each municipality in this ATO considered the total cost of waste collection per capita as input, the tons of unsorted waste as bad output and the tons of sorted waste as output. In addition, we incorporated two external factors, SIZE and RICHNESS, to refine the comparisons and define the conditional directional distances to the efficient frontier, thus accounting for municipality heterogeneity within the waste *community*. This approach allowed us to calculate viable eco-efficiency targets for each municipality and estimate gaps between the original input, bad output and output, facilitating meaningful comparisons.

The results revealed that there is room for a general increase in efficiency within the analyzed waste community. In more detail, we observed an *inverted U-shaped* relationship between inefficiency in output production (tons of sorted waste) and municipality *size*. Initially, as municipality size increased, there was an increasing trend in inefficiency (indicating *decreasing* returns to scale). However, beyond a certain threshold, we observed a decreasing trend in inefficiency for larger municipalities (indicating *increasing* returns to scale). Conversely, economic development showed the opposite trend with respect to municipality size, defined by a *U-shaped relationship* between inefficiency in output production (tons of sorted waste) and *economic development*. Initially, as economic development increased, inefficiency decreased (indicating *increasing* returns to scale). However, beyond a certain point, inefficiency in output production increased for municipalities with higher values of economic development (indicating *decreasing* returns to scale).

These results underscore the significance of size and economic development in achieving viable eco-efficiency within the analyzed MW collection community. Municipalities could improve their performance by achieving an optimal size that allows them to benefit from economies of scale. Policymakers and waste management authorities should consider these findings when designing and implementing waste collection policies regarding tons of sorted waste.

To enrich our analysis, we explored two case studies of Ciampino and Guidonia Montecelio, respectively, analyzing their viable eco-efficiency targets based on benchmark municipalities of comparable size and development within the waste community. Our findings identified Albano Laziale as the most comparable benchmark for Ciampino, and Velletri for Guidonia Montecelio. Through this case analysis, we uncovered that municipalities could achieve efficiency despite higher costs by collecting fewer tons of unsorted waste (BY) in comparison to sorted waste (Y), thereby prioritizing a high Y and minimal BY.

It is important to note some limitations of the present study, including the exclusion of certain municipal characteristics due to the limited sample size. Additionally, the presence of outliers may have skewed the results. Future analyses could adopt more robust methodologies, such as those presented in^38^, to mitigate these issues. Furthermore, our study excluded analysis of social change, which is fundamental to advancing MW management within a CE perspective^65^. Moreover, the impact of education and youth presence in cities was not explored, despite the significance of these factors in driving sustainable transitions, as suggested by^66^. Importantly, the sustainable and efficient development of MW collection may rely on participatory and collaborative efforts.

Taking a first step in this direction, the Latium region and relevant authorities must promptly establish collaborative government bodies, as mandated by Law 23 December 2009 n. 191^67^, (art. 2 paragraph 186-bis). These bodies would bring together municipalities, urban waste managers, collection service managers and regional governors to coordinate and improve waste management activities collaboratively. Alarmingly, according to the monitoring carried out by Re-Open SPL^68^, Latium stands as the sole Italian region yet to establish these government bodies in its ATOs. The establishment of these bodies is imperative for achieving the identified efficiency targets at an operational level. As evidenced in the case studies of Guidonia and Ciampino, whose benchmarks share the same manager, direct communication between managers and policymakers may facilitate operational improvements and the sharing of best practices.

One strength of the present analysis is its rich, micro-level investigation of a single *community* (ATO-RC). However, this focus also represents a limitation of the work. To address this, future research should aim at extending the analysis. The proposed approach is general and adaptable and may be applied to municipalities across Italy or Europe. It may also be expanded to meso (regional) or macro (country) levels of analysis.

Of note, while the present analysis primarily considered external economic (RICHNESS) and environmental (SIZE) factors in the eco-efficiency framework, it overlooked the significant social implications of waste collection services. Future research aimed at translating *viable* targets into *sustainable* targets (considering all three dimensions of sustainability) should incorporate social indicators such as job creation in waste management, equitable access to waste collection services (both door-to-door and non-door) and social acceptance of door-to-door waste collection policies (i.e., the temporary reserve of part of a house or apartment block for waste collection at appropriate times) or roadside collection (i.e., waste taken to roadside containers). Additionally, social policies such as recycling incentive programs and public awareness campaigns could be integrated into the analysis to provide a more comprehensive understanding of waste management practices.

In conclusion, we highly recommend that municipalities integrate waste collection with other sectors to significantly improve waste management performance at a community level. By aligning waste collection systems with sectors such as energy, water and transport, municipalities may leverage synergies to improve efficiency and reduce environmental impacts and costs associated with waste management. Municipalities should therefore promote and facilitate cross-sectoral cooperation and coordination, drawing inspiration from successful models such as energy communities. The establishment of platforms, networks and partnerships may facilitate the exchange of information, resources and best practices, enabling citizens to actively contribute to sustainability goals^69^. Furthermore, the quality and effectiveness of waste collection services may be improved through innovation and the digitization of waste management systems. These advancements would not only optimize the waste collection process, but they would also improve monitoring and evaluation capabilities, ensuring transparency and accountability while providing valuable feedback and guidance to both users and operators.

### Supplementary Information

Supplementary Information.

## Data Availability

The datasets used and/or analyzed for the present study are available from the corresponding author upon reasonable request.
