# Supplementary material for: Viable eco-efficiency targets for waste collection communities
Source: Sci Rep. 2024 Jul 1;14:15038. doi: 10.1038/s41598-024-66077-y (PMC11217406; doi:10.1038/s41598-024-66077-y)
Supplement: Supplementary file 1 — Supplementary Information. [file 41598_2024_66077_MOESM1_ESM.docx]

**Appendix A**

The PCA analysis identified the first two principal components, which collectively explained 76% of the original variance. Table A1 displays the correlations between these principal components and the external factors considered.

The first principal component (PC_1_), which was strongly correlated with the size-related variables (i.e., population, touristic capacity, territorial dimension), was termed SIZE and designated as our first external variable (Z_1_). The second principal component (PC_1_), highly correlated with income per capita, was labeled RICHNESS and included in our analysis as our second external variable (Z_2_). Hence, the directions each municipality should follow to approach the efficient frontier (or frontier of best practices), were determined by Z_1_ and Z_2_. Z_1_ ranged from a minimum value of -3.66 to a maximum of 3.81, with increases corresponding to SIZE. Z_2_ ranged from -1.76 to 2.98, with increases correlating with RICHNESS.

|  | ***PC1*** | ***PC2*** | ***PC3*** | ***PC4*** | ***PC5*** |
| --- | --- | --- | --- | --- | --- |
| **Log population** | 0.93 | -0.06 | 0.09 | 0.2 | -0.29 |
| **Log tourist capacity** | 0.91 | 0.16 | -0.05 | 0.27 | 0.26 |
| **Log income per capita** | 0.24 | 0.95 | -0.14 | -0.16 | -0.05 |
| **Log km^2^** | 0.64 | -0.34 | -0.64 | -0.23 | 0 |
| **Altitude** | -0.76 | 0.13 | -0.54 | 0.33 | -0.06 |

*Table A1 Correlations between principal components and the external factors considered.*
